# Could it be colorectal cancer? General practitioners’ use of the faecal occult blood test and decision making – a qualitative study

**DOI:** 10.1186/s12875-015-0371-1

**Published:** 2015-10-26

**Authors:** Cecilia Högberg, Eva Samuelsson, Mikael Lilja, Eva Fhärm

**Affiliations:** Department of Public Health and Clinical Medicine, Unit of Research, Education and Development - Östersund, Umeå University, Umeå, Sweden; Department of Public Health and Clinical Medicine, Umeå University, Umeå, Sweden

**Keywords:** Colorectal neoplasms, Diagnostic techniques and procedures, Occult blood, General practitioners, Primary health care, Qualitative research

## Abstract

**Background:**

Abdominal complaints are common reasons for contacting primary care physicians, and it can be challenging for general practitioners (GPs) to identify patients with suspected colorectal cancer (CRC) for referral to secondary care. The immunochemical faecal occult blood test (iFOBT) is used as a diagnostic aid in primary care, but it is unclear how test results are interpreted. Studies show that negative tests are associated with a risk of delayed diagnosis of CRC and that some patients with positive tests are not investigated further. The aim of this study was to explore what makes GPs suspect CRC and to investigate their practices regarding investigation and referral, with special attention on the use of iFOBTs.

**Method:**

Semi-structured individual interviews were conducted with eleven purposely selected GPs and registrars in Region Jämtland Härjedalen, Sweden, and subjected to qualitative content analysis.

**Results:**

In the analysis of the interviews four categories were identified that described what made the physicians suspect CRC and their practices. *Careful listening—with awareness of the pitfalls:* Attentive listening was described as essential, but there was a risk of being misled by, for example, the patient’s own explanations. *Tests can help—the iFOBT can also complicate the diagnosis:* All physicians used iFOBTs to various extents. In the absence of guidelines, all found their own ways to interpret and act on the test results. *To refer or not to refer—safety margins are necessary:* Uncertainty was described as a part of everyday work and was handled in different ways. Common vague symptoms could be CRC and thus justified referral with safety margins. *Growing more confident—but also more humble:* With increasing experience, the GPs described becoming more confident in their decisions but they were also more cautious.

**Conclusions:**

Listening carefully to the patient’s history was essential. The iFOBT was frequently used as support, but there were considerable variations in the interpretation and handling of the results. The diagnostic process can be described as navigating uncertain waters with safety margins, while striving to keep the patient’s best interests in mind. The iFOBT may be useful as a diagnostic aid in primary care, but more research and evidence-based guidelines are needed.

## Background

Abdominal and bowel complaints are common reasons for contacting general practitioners (GPs) and are mostly caused by benign conditions [1, 2]. However, it is important to identify serious diseases such as colorectal cancer (CRC). Worldwide, CRC is the third most common cancer in men and the second most common in women [3]. In Sweden, it is the third most common cancer in both sexes, and approximately two-thirds of the patients who are diagnosed with CRC have initially consulted a primary care physician [4–6]. In spite of this, CRC is a rare diagnosis for the average GP in Sweden, who can expect to encounter fewer than one new case a year [7].

CRC often presents with vague symptoms, and this can cause a delay in diagnosis, which is probably associated with poorer outcome [8]. It is thus important to identify ways to make an early diagnosis.

Faecal occult blood tests (FOBTs) are used for CRC screening, and many studies have reported on this [9]. They are also used as diagnostic aids [10–12], but there is little evidence supporting this use. The tests could potentially be helpful [13, 14], but the risk of delayed diagnosis increases with false negative test results [6, 15]. It is unclear how the test results are interpreted and applied in everyday clinical practice, and studies have shown that many patients with positive tests are, for unknown reasons, not investigated further [10, 11].

Recommendations for the use of FOBTs in clinical practice vary. Sweden has no national guidelines regarding the use of FOBTs or the investigation of suspected CRC. In Denmark, FOBTs are recommended for use in secondary care for patients with changed bowel habits when the sigmoidoscopy results are normal, and in Ontario, Canada, they are recommended for use in primary care for patients with a low suspicion of CRC and no rectal bleeding [16, 17]. In the United Kingdom it is recommended to offer FOBTs in primary care in certain cases in adults without rectal bleeding [18]. New guidelines for suspected CRC recognition and referral were published in the United Kingdom in June 2015, and there is an ongoing discussion about the use of the FOBT as a diagnostic aid in primary care [19].

As bowel symptoms are common and the symptoms of CRC are often vague, it can be challenging for GPs to decide which patients to refer for further investigation. There are also questions as to the use and usefulness of FOBTs for investigating suspected CRC in primary care. The aim of this study was to explore what makes GPs suspect CRC and to investigate their practices regarding investigation and referral, with special attention on the use of FOBTs.

## Methods

Semi-structured individual interviews were conducted with GPs and registrars in Region Jämtland Härjedalen, a sparsely populated region in northern Sweden that includes an area somewhat larger than the Netherlands and that has around 127.000 inhabitants. There are primary health care centres spread throughout the region, with one hospital centrally located in the only town. Primary care plays a gatekeeper role in non-acute illnesses. Rectoscopies are performed in primary care at all of the region’s health care centres, as are point-of-care FOBTs [6]. As in the rest of Sweden, GPs refer patients directly for bowel imaging; the referrals are all sent to the hospital’s endoscopy or radiology departments. There is no CRC screening program. The faecal test that is used is an immunochemical FOBT (iFOBT), and the usual practice is to test three samples.

The participants were purposely selected. In the region 63 GPs and 26 registrars were eligible for the study. They were sorted into eight groups with respect to gender, length of professional experience, and distance from the workplace to the hospital. From each group one person was drawn. These persons were sent an invitation letter with information about the study and then contacted by telephone for further information; if they wanted to participate an interview was arranged. They were informed that the aim of the study was to explore what made the GPs suspect CRC and their practice, not to judge the way they handled the patients, and that there were no right or wrong responses. A topic guide was constructed before the interviews and then revised and supplemented after the first and second interviews. The interviews were performed face-to-face, audiotaped, and transcribed verbatim by one of the authors (CH). Each interviewee read their transcribed interview and was invited to make corrections and additions, none of them made any changes.

Initially, six GPs and two GP registrars were invited. Two of these declined to participate, referring to lack of time, and in their places two others were invited and interviewed. The interviews lasted from 20 to 49 (average 37) minutes. After analysing these eight interviews, we saw a need for additional interviews to confirm our findings; accordingly, we chose, invited and interviewed another three GPs. By the eleventh interview, data saturation seemed to be reached. To confirm this, supplementary telephone interviews were conducted with two of the first six GPs. Informed consent was obtained from all participants. Four of the GPs had less than ten (mean 4.5) years and five had more than ten (mean 25) years of experience of work as a GP. Five of the GPs worked at health care centres with a distance to the hospital of less than 25 (mean 9) kilometres, and four at health care centres with a distance of more than 25 (mean 67) kilometres. The registrars had a mean professional experience of 3.5 years after graduation and both had their workplaces less than 25 km from the hospital.

This study used qualitative content analysis with an inductive approach as described by Graneheim and Lundman [20]*.* The transcribed interviews were read thoroughly by all of the authors. The analytical process included naïve reading of the interviews to obtain a sense of the whole plus interpretation of the latent content of the interviews. CH first extracted and condensed meaning units for five of the interviews, then all authors discussed the result and agreed on further approach. Two of the authors then separately coded each interview; CH coded all the interviews and the other authors coded three or four interviews each. Categories were identified, and all codes were sorted into these, refining the categories during this process. A consensus on data saturation, codes, categories, and analysis was reached through group discussions that involved all authors. The categories are presented in the results section and are illustrated with quotes that are marked with individual numbers for each participant. Examples of the analytical process are presented in Table 1.

**Table 1:** An example of the analytical process used to process the interview information

Meaning unit	Condensed meaning unit	Code	Category
I assume there’s also a certain degree of intuition involved. Like when something is not quite right with a person I’ve seen before.	Intuition involved when a patient has been seen before.	Intuition involved.	Careful listening—with awareness of the pitfalls
It (iFOBT^a^) could be important because it can back up my theory if I already, based on other symptoms and so on, have an idea.	IFOBT results can back me up if I, based on other factors, have a general idea.	IFOBT can back up a decision to investigate or not.	Tests can help—the iFOBT can also complicate the diagnosis
If I don't feel quite certain about an IBS^b^ diagnosis, I refer for bowel imaging.	Bowel imaging if the IBS diagnosis feels uncertain.	Bowel imaging if an uncertain IBS diagnosis.	To refer or not to refer—safety margins are necessary
Perhaps I can keep a cool head for longer now, if I think it looks harmless.	Keep a slightly cooler head now if it looks benign.	Keeping cooler head now.	Growing more confident—but also more humble

^a^Immunochemical faecal occult blood tests

^b^Irritable bowel syndrome

Ethical approval was obtained from the Regional Ethical Review Board, Umeå (Dnr 2013/326–31).

## Results

When we analysed the interviews in an effort to determine what makes GPs suspect CRC and to identify their practices in further investigations, four categories were identified. Each is described below.

### Careful listening – with awareness of the pitfalls

The importance of attentive listening to the patient and of taking a careful, thorough medical history was stressed, and the GP’s background knowledge of the patient was taken into consideration.

The interviewees described using their basic knowledge of the manifestations of CRC to evaluate the patient’s history. Symptoms that were new to the patient aroused suspicion. Rectal bleeding and changes in bowel habits were considered important factors, but vague symptoms, anaemia, and clinical findings could also elicit suspicion. Age, family history, and the patient’s previous help-seeking behaviour and anxiety could influence.

*(What causes you to be suspicious?)* “Traces of blood in the stool could be one thing. Also, that the patient, perhaps not as specific, feels unwell or has lost weight. … Changed bowel habits of course. … Sometimes, nonspecific abdominal pain.” (GP9)

“The patient feels different in some way.” (GP3)

If the patient regarded his or her bowel habits to be changed, they were considered to be changed.

“It’s the patient that knows how things were. … If something brings the patient to me because he or she feels that something is different, then there is in fact a change.” (GP2)

However, careful listening to the patient’s history carried a risk of being misled by the patient’s own explanations of his or her symptoms. A history of menorrhagia with anaemia, haemorrhoids, or irritable bowel syndrome (IBS) could also be misleading. Continuity of care could be helpful but also a risk.

“Sometimes, patients have their own explanations, which may act as a kind of smokescreen. … The explanation they come up with can ultimately delay things.” (GP3)

“This group of patients that are nervous and contact us very often, they live a little dangerously, because if they did develop something malignant, then there is a big risk that things would be delayed.” (GP6)

With polysymptomatic patients, it could be difficult to grasp what was important to listen to:

“Middle-aged and older patients with a lot of symptoms who perhaps add, in passing, that something feels a bit different. Sorting through this plethora of symptoms, well, that can sometimes be difficult.” (GP 8)

### Tests can help - the iFOBT can also complicate the diagnosis

When investigating possible CRC, all physicians used the iFOBT and other laboratory tests. All discussed the limitations of iFOBTs, but their handling of the test results varied. Some found the iFOBT to be an important aid, while others were doubtful about its usefulness.

They all said that they initially ordered a standard battery of laboratory tests with personal variations. Anaemia was described as a significant finding that was important to investigate further.

The iFOBT results were considered to be easiest to evaluate when all three samples of a set were positive or negative. An iFOBT with three positive samples generally resulted in a referral and was thought to perhaps quicken the referral process and be helpful in prioritising at the hospital.

“If my clinical findings support me in taking things further, and I also have three positive iFOBTs, then I think this strengthens the investigation and can perhaps speed up the referral as well.” (GP1)

Three negative samples could support watchful waiting. Negative iFOBTs were not thought to be conclusive, and it could be difficult to determine whether these were sufficient to exclude CRC. There were also situations when there was conflicting information from the medical history and the iFOBT results.

“But, in situations where I’m wavering, it might support the theory; I did actually have three negative iFOBTs.” (GP2)

“When the iFOBTs are negative but the patient is experiencing symptoms, I still move forward with the investigation.” (GP4)

When just one of three samples was positive, there was a grey area. Some regarded this as a positive test and referred the patient for bowel imaging, while others were more hesitant and sometimes repeated the test, taking another three samples. If repeated tests were negative and the patient’s history seemed benign, the physician could decline to refer the patient, who was subsequently followed at the healthcare centre.

“Fact is, I always act on a positive iFOBT, even when I am convinced the symptoms are functional. I take these tests for a reason.” (Registrar2)

(*one of three samples positive*) “Then it’s more difficult. Yes, I redo it. Yes. And, if there’s then a series of three negatives, well then I consider it a negative, yes. Naturally, it’s also the problems, the symptoms that determine if you can, sort of, let it go.” (GP6)

With a history of rectal bleeding, some often used iFOBT to confirm the bleeding, while others found this unnecessary.

“Many times, I think I’ve done the iFOBT to verify, to have it in the records as well, that this is really the case.” (GP1)

“If there is visible blood, then I naturally don’t do any iFOBTs.” (GP7)

The interviewees described being generous with the use of rectoscopy; with a history of rectal bleeding it was performed as a rule. Many thought that it was difficult to decide when rectoscopy findings were sufficient to explain rectal bleeding, irrespective of the iFOBT results. With positive iFOBT results, findings of haemorrhoids were sometimes considered to be a sufficient explanation. Negative iFOBTs could sometimes reinforce the decision not to refer for bowel imaging when there was a history of rectal bleeding and findings of haemorrhoids, especially in younger patients.

“If I have positive iFOBTs, then I’d like to know why this is the case … and in those cases a rectoscopy may suffice, if I find something that’s bleeding there.” (Registrar1)

There were different opinions on iFOBTs’ usefulness. While some found them to be of great help, others found them not especially helpful.

“Yes, they’re of huge help, yes. … They’re of crucial importance.” (GP6)

“Yes, they serve as an indication.” (GP8)

“In reality, they’re not so useful. … They don’t help me very much, I’m hesitant to the usefulness of iFOBT.” (GP5)

### To refer or not to refer - safety margins are necessary

The interviewees described their efforts to make clinical judgements that were plausible, and in which they felt reasonably secure. They wanted better communication both with and within secondary care and strived to keep their patients’ best interests in mind.

Uncertainty was described as a part of everyday work. Patients with recurrent and vague symptoms could be the most difficult to handle.

“I think, after all, this is my job! This is what I get paid to do, so I have to make the assessment and take responsibility for it. It’s up to me to harbour the uncertainty.” (GP2)

‘The real difficulty, I think, is actually people with vague stomach problems, perhaps with IBS, who come to us from time to time… when have things changed so much, and when has so much time passed that it’s time to move on again?” (GP2)

To handle uncertainty they thought it was helpful to discuss cases with their colleagues at the health care centre or to ask specialists in secondary care for their opinions. It could also be helpful to reflect upon things for some time before deciding upon a course. Involving the patient in the referral decision was considered to be important. Especially when there was some uncertainty, they thought it important with a dialogue and to come to a consensus with the patient, all without passing on the feeling of uncertainty.

“This is probably the hardest part. When things are vague, when to leave it. The conversation with the patient is really important in order to sense where he or she stands psychologically in all this.” (GP4)

In general, the GPs considered themselves to be generous with referrals, but they also thought that the referrals should have reasonable grounds.

‘The level of investigation must have a safety margin … Otherwise, we’re too restricted in our investigations.” (GP6)

“Most of what we investigate turns out to be nothing. … You have to draw the line somewhere and keep a cool head and wait a while.” (GP7)

Once having decided to refer, the physicians had to choose between a referral for a radiological examination or an endoscopy. As resources for colonoscopy were limited, referral for this could involve advocating for their patients. Many described experiences of insufficient information from the hospital regarding which patients to refer to what department and about current (sometimes long) waiting times. They also told of their concern about increasing demands from secondary care that tests should be carried out in primary care before referral.

“Sometimes you find a haemoglobin level that is so low that you don’t want to investigate things at a primary care level because you know how long it takes there, and then you send the patient to the hospital because you think they will get admitted, but they don’t always.” (GP3)

There were worries about colonoscopy and laxation being trying for the patients. Here, too, long distances to the hospital could be a problem. The interviewees engaged district nurses to help with laxation and travel arrangements, and sometimes they arranged for hospital care.

“In particular, many older patients would benefit from being admitted to have purging done at the hospital.” (GP5)

When the decision was not to refer, the doctors described using different levels of safety netting. Time was considered an important tool.

“If you feel quite certain, you can leave it very open, for example, say something like ‘get in touch if you need to’. If you feel there are uncertainties, perhaps give the patient a time frame, like ‘get in touch no later than this time, or if exactly this or that happens’.” (GP1)

### Growing more confident – but also more humble

With increasing experience, the GPs described being more confident in making decisions but also becoming more humble.

They described feeling more secure about not examining every symptom in detail, and learning to harbour uncertainty and live with the fact that nothing was certain.

“Deciding whether to investigate or not, for example. And, how to follow up. I’ve gotten used to it over the years, and it’s not a big problem. I can make a decision pretty well and then let it go without it bothering me.” (GP7)

Gut feelings were considered to be based on experience, and so they changed over time. With greater experience, it could also be easier to see whether patients diminished their problems.

“Perhaps you understand people better with time, you understand that some perhaps play down their symptoms because they’re scared of what it could be, and that you somehow see through this better with time.” (GP6)

The interviewees also noted that growing knowledge and experience did not always lead to greater certainty. Instead, they described becoming more cautious, with a greater awareness of the risk of pitfalls, and perhaps being more generous with referrals for bowel imaging.

“You don’t always feel more confident just because you have more information. … I’ve become more uncertain about things like iFOBT, for example. … I used to think those tests were a lot more help than I do these days.” (GP4)

The GPs described becoming less concerned about what others thought about their referral decisions and also more humble. The patients were their focus. Nevertheless, they valued dialogue with their colleagues at the hospital and wished for better communication.

“I’ve worked for quite a few years … there’s no work prestige involved. … I don’t really care if there is someone at the other end that laughs at my referral … it’s not my problem.” (GP9)

“I still think it would be better if I could discuss things a bit more with the hospital.” (GP3)

All of the GPs recounted their personal experiences with patients that turned out to have CRC. In some cases, it had been easy to decide how to act, while delays were described in other cases. A menstruating woman with anaemia was cited as one example of a situation that resulted in a delay, and a second example was a patient who did not want to be referred.

## Discussion

The findings from this study showed that the interviewees, in the absence of guidelines or consensus, all found their own ways to interpret and handle the iFOBT results—these were perceived as sometimes being helpful and sometimes complicating the matter. A positive iFOBT result could reinforce the decision to refer a patient for further investigation, and negative results were not entirely trusted. The iFOBT results were considered to have less weight than the patient´s history. This is in line with the low positive predictive value of this test and an example of probabilistic reasoning [6, 21].

Positive iFOBTs were used as a way to emphasise the need for bowel imaging and were thought to (perhaps) help secondary care prioritise patients. Therefore an iFOBT was sometimes ordered also if there was a history of rectal bleeding. Practical reasons for ordering laboratory tests, among many other factors, were observed in an earlier study [22].

As CRCs may bleed intermittently, it is reasonable to consider the iFOBT as positive if one of three samples is positive and then to refer for bowel imaging. However, in this study, there were divergent views on what further action should be taken in these cases. Some patients were followed in primary care and never referred to secondary care, which could at least partly explain findings in earlier studies that patients with positive FOBTs were not investigated further [10–12]. There could be several possible reasons for this; for example medical histories considered to be benign, GPs’ earlier experiences of many false positive tests, haemorrhoids found at rectoscopies, patients unwilling to be referred for bowel imaging, poor availability of and long waiting times for bowel imaging, as well as the absence of guidelines. After a repeated test that was negative the GP weighed the medical history against the test results and could come to the decision not to refer.

Listening carefully to the patients also means listening to the patient´s potential smoke screens and perceived diagnoses, which can mislead the physician [23]. There is thus an underlying conflict in that the physician must at the same time listen to the patient and avoid being misled by the patient. Especially with polysymptomatic patients and those with vague symptoms, it can be a challenge to identify those who need further investigation of suspected CRC [24].

‘Change in bowel habits’ was a symptom all mentioned. This expression has a connotation of suspected CRC, but to our knowledge there is no commonly accepted definition of what this means [25]. Many of the participants in this study described that if a patient considered their bowel habits to be changed, then the physicians, too, considered them changed. Thus the patients individually determined the definition.

Continuity of care is generally thought of as a positive factor [26–28]. However, it can also include a risk of delay [28–30]. In this study, continuity and earlier knowledge of the patient was mostly considered helpful, but patients with frequent consultations were thought to be at risk of delay.

Concerns about long waiting times, increased or changed demands from secondary care, and the desire for better communication with secondary care consultants, all of which were described in our study, have also been reported from other countries [24, 31, 32]. It seems likely that the secondary care consultants would also appreciate better communication [33].

Deciding whether to refer a patient includes management of uncertainty, and this is an essential part of working in primary care [34]. The interviewees described different ways of dealing with this: ordering laboratory tests, seeking more knowledge, involving the patient in the decision, taking time to reflect, safety netting, referring with safety margins, discussions with colleagues, and asking for a second opinion. With increasing experience, decision-making seemed easier for the GPs in our study, which is in line with the findings of a study in Finland [35]. However, the increased experience did not seem to result in less uncertainty; instead, personal experiences of the difficulties in choosing the right patients to refer appeared to lead to greater cautiousness and humility.

### Strengths and limitations

The physicians in this study were diverse in terms of gender, work experience, and the location of the health care centres; only two physicians who were invited declined to participate, referring to lack of time. The study participants confirmed the contents of their transcribed interviews. We aimed to use well-structured methodology in the analysis in order to add to the credibility of our study.

The number of physicians that were interviewed and the geographic area that was covered was limited. However, the information gleaned from the interviews was plentiful, and we have aimed to describe the context in which the study took place to make transferability judgements possible.

### Implications for clinical practice and future research

Our study results illustrate the difficulties in diagnosing a low-incidence serious disease that presents with common symptoms. The use of the iFOBT as described by the participants illustrates the consequences of using a test in a population for which it has not been evaluated. The iFOBT could potentially be useful as a diagnostic aid in primary care, but more research is needed. Our study also shows the need for evidence-based guidelines and improved communication between GPs and consultants.

## Conclusion

Careful listening to the patient’s history was essential for prompting suspicion of CRC and for making a decision about which patients to refer. The iFOBT was used frequently as support, but there was a considerable variation in the handling of the results. The diagnostic process can be described as navigating uncertain waters with safety margins, while striving to keep the patient’s best interests in mind.

## Abbreviations

CRC: Colorectal cancer
GP: General Practitioner
FOBT: Faecal occult blood test
iFOBT: Immunochemical faecal occult blood test
